# Active and adaptive case finding to estimate therapeutic program coverage for severe acute malnutrition: a capture-recapture study

**DOI:** 10.1186/s12913-019-4791-9

**Published:** 2019-12-16

**Authors:** Sheila Isanaka, Bethany L. Hedt-Gauthier, Halidou Salou, Fatou Berthé, Rebecca F. Grais, Ben G. S. Allen

**Affiliations:** 1 Department of Nutrition and Global Health and Population at Harvard School of Public Health, Boston, USA; 2 Department of Research, Epicentre, Paris, France; 3 Department of Global Health and Social Medicine (Harvard Medical School), Boston, USA; 4 Department of Biostatistics (Harvard School of Public Health), Boston, USA; 5 Epicentre Niger, Maradi, Niger; 6 Epicentre Nigeria, Sokoto, Nigeria; 7 Technical Rapid Response Team and International Medical Corps, Washington, DC USA

**Keywords:** Active and adaptive, Case finding, Capture recapture, Coverage, Severe acute malnutrition, SQUEAC, Therapeutic feeding program, Nigeria, Community-based management of acute malnutrition

## Abstract

**Background:**

Coverage is an important indicator to assess both the performance and effectiveness of public health programs. Recommended methods for coverage estimation for the treatment of severe acute malnutrition (SAM) can involve active and adaptive case finding (AACF), an informant-driven sampling procedure, for the identification of cases. However, as this procedure can yield a non-representative sample, exhaustive or near exhaustive case identification is needed for valid coverage estimation with AACF. Important uncertainty remains as to whether an adequate level of exhaustivity for valid coverage estimation can be ensured by AACF.

**Methods:**

We assessed the sensitivity of AACF and a census method using a capture-recapture design in northwestern Nigeria. Program coverage was estimated for each case finding procedure.

**Results:**

The sensitivity of AACF was 69.5% (95% CI: 59.8, 79.2) and 91.9% (95% CI: 85.1, 98.8) with census case finding. Program coverage was estimated to be 40.3% (95% CI 28.6, 52.0) using AACF, compared to 34.9% (95% CI 24.7, 45.2) using the census. Depending on the distribution of coverage among missed cases, AACF sensitivity of at least ≥70% was generally required for coverage estimation to remain within ±10% of the census estimate.

**Conclusion:**

Given the impact incomplete case finding and low sensitivity can have on coverage estimation in potentially non-representative samples, adequate attention and resources should be committed to ensure exhaustive or near exhaustive case finding.

**Trial registration:**

ClinicalTrials.gov ID NCT03140904. Registered on May 3, 2017.

## Background

Program coverage is a measure of how many individuals in need are receiving treatment or an intervention. It is an important indicator to assess the performance of public health programs and is essential to inform program planning and prioritization of limited resources. Coverage, combined with program effectiveness, is critical to assess how many of those in need are accessing treatment or prevention activities and achieving the desired outcome.

In the management of severe acute malnutrition (SAM), several practical methods for treatment coverage estimation have been proposed [1] that identify cases using active and adaptive case finding (AACF). AACF is an informant-driven sampling method that yields a sample of individuals who possess specific characteristics and have been referred by others, starting with a “seed” or key informant(s) to begin the referral chain [1, 2]. Similar methods have commonly been used when sampling hard-to-reach populations such as injection drug users [3] or men who have sex with men [4]. When sampling children with SAM, AACF has two advantages: it is active and therefore does not rely on cases self-presenting as in central point sampling, thus avoiding cases not arriving due to stigma associated with the illness, distance or other factors [5]; and it is efficient as only houses of suspected cases, not all houses, in a sampling area are visited. AACF is particularly suitable for conditions with symptoms that can be visibly identified and that are rare and therefore require a larger sampling area in order to reach an adequate sample size. However, as this method can yield a non-representative sample, AACF should be exhaustive or nearly exhaustive to yield valid estimates of coverage [1].

Although practical guidelines have been proposed to indicate when sample exhaustion has been reached during AACF [1, 2], there is uncertainty around whether the method can ensure an adequate exhaustivity in operational settings [6]. Debate surrounding the practical validity of the case finding method thus remains. To inform the continued use of AACF in the estimation of SAM treatment coverage, we assessed the exhaustivity of AACF to identify SAM cases in northwestern Nigeria.

## Methods

### Study setting

This study was conducted in the Wamako Local Government Area in Sokoto State of northwestern Nigeria in 2017. The region is characteristic of the rural Sahel and has a stable population with a high burden of acute malnutrition (global acute malnutrition: 10.4, 95% CI: 7.5, 14.2% in 2015 [7]). From 2013 to 2017, International Medical Corps supported the Sokoto State Ministry of Health to deliver treatment of uncomplicated SAM at five outpatient centers, with community surveillance and outreach teams in approximately 430 villages (average village size: 483 people) [8].

### Study design

For this study, we defined sensitivity as the probability of a sampling method to correctly identify a child in the community that has SAM or is recovering from SAM. We assessed the sensitivity of AACF using a capture-recapture design [9, 10]. Capture-recapture designs were first used in ecological studies to estimate animal populations [10] and have more recently been applied to assess the total case population of health conditions using two independent sources, such as two disease registers [10, 11]. In a capture-recapture study, two case finding methods are used to determine the size of the total case population, and with that information, the sensitivity of each case finding method can be estimated [9]. In this study, AACF was compared to a census method where all households were visited and all children 6–59 months screened on sequential days. While often considered to be a gold standard, the census method may miss cases, for example due to routine absence from the household on the day of recapture. The capture-recapture study design does not require that either case finding procedure find 100% of cases to estimate the total number of cases in the study population or method-specific sensitivity [9]. However to be valid, the capture-recapture study must adhere to five assumptions: closed population; ability to perfectly match cases captured in both methods; in both methods, perfect classification (perfect diagnosis of SAM and coverage status); within a method, any child with SAM has equal probability of capture; and independence of capture between methods [9–11] (Table 1).

**Table 1:** Descriptions of the assumptions underlying AACF and study procedures to reduce potential violations

*Assumption*	*Description*	*Study procedure*
Closed population	The population sampled using both methods is the same. In this case the same households are considered during each day of case finding, there are no absent cases on either day and only residents of the village are considered.	- The same guide was used to ensure the same village boundaries were used on both days. - The second day of case finding was performed immediately after the first day limiting the possibility of movements of people in between samples. - On the first day of sampling all cases found were encouraged to remain at home the following day and the village leader was also requested to communicate the same message to limit the amount of absent cases on the second day. - Known market and treatment days were avoided for both first and second days of case finding, and on the first day village leaders were asked whether there was likely to be significant movements of people (to a market for example). - All cases were proven (by asking mother and double checking with village leader, or other informant) to be from the selected village.
Ability to perfectly match cases captured in both methods	Cases found in both samples can be reliably identified and there is certainty when a case is only found in one sample.	- Four matching variables (first and second name, age and sex of child) were collected during both samples.
Perfect classification (Perfect diagnosis of SAM and coverage status in both methods)	Cases are correctly identified and there is no over or under diagnosis of cases.	- A rigorous, clear and context specific case definition was developed prior to the study. - Surveyors were trained in case identification, including screening for SAM by mid-upper arm circumference (MUAC) measurements and edema testing, and screening for recovering cases by asking carers to present sachets of ready-to-use therapeutic food (RUTF) or clearly explain treatment schedule.
Equal probabilityof capture within a method	All cases should have the same chance of being found including very sick or hidden children.	- If any suspected cases were away at the time of the household visit teams returned at the end of the day in case they had returned. - The survey team conducted a quick census at each household visited to ensure there were no children sleeping or being hidden. - If children were not present in the household but were nearby they were found by the survey team.
Independence of capture between methods	When a child is found in the first sample, this does not increase (positive dependence) or decrease (negative dependence) the likelihood of being found in the second sample.	- Assured given systematic sampling conducted during census method.

### Sample size

Current operational guidance on the use of capture-recapture studies to validate SAM case finding recommends that the estimated number of cases found in both samples be greater than seven and the number of total cases found across both samples be greater than the estimated SAM population [9, 12]. A priori*,* we estimated AACF would capture 40% of SAM cases (sensitivity_AACF_ = 40%) and that a census would capture 80% of SAM cases (sensitivity_census_ = 80%). This would require 24 SAM cases to exist to meet the first condition. Assuming an average village size of 483 [8], a SAM prevalence of 2.7% [7] and the proportion of children aged 6–59 month to be 20% of the population [7], nine villages were estimated to be necessary to identify 24 SAM cases. Given the time and resources available, 15 villages were ultimately sampled to be sure that the minimum sample size would be reached for the first condition above.

### Study procedures

#### AACF method

Prior to case finding, a SAM screening definition was developed using qualitative methods [2] [see Additional file 1]. Semi-structured interviews were first conducted in four villages. An interview guide was used to identify context-specific terms related to SAM, which were triangulated and used to devise a screening definition. This screening definition was then iteratively tested and revised with new information over three days until no new information was found. The resulting screening definition included terminology in two local languages (Hausa and Fulani) to describe the signs and symptoms of SAM as well as associated illnesses. Stigmatizing terms were identified to ensure they were avoided, and teams were aware if used by informants. Information on local beliefs about the etiology, health-seeking behaviors and the types of individuals with knowledge about children with SAM were also collected. This additional information was collected to allow enumerators to target individuals during case finding that would be more knowledgeable about the location of SAM cases.

During case finding, the context-specific screening definition iteratively developed for the study, as well as photos of malnourished children and packets of ready-to-use therapeutic food (RUTF) used for the treatment of SAM, were presented in each sampled village to help orient key informants towards suspected SAM or recovering cases. Key informants included traditional birth attendants, village leaders, caregivers, grandmothers, traditional healers, community nutrition volunteers, children and health center staff. The houses of all suspected cases were visited for individual evaluation. In each household, a brief household interview was conducted to ensure no child aged 6–59 months was sleeping or absent. All children present were assessed for SAM, defined as mid-upper arm circumference (MUAC) < 11.5 cm and/or bilateral pitting edema (Table 2). To identify recovering cases in the household, caregivers were asked if any child was undergoing treatment for SAM. RUTF sachets were presented to confirm enrollment. All identified SAM and recovering cases were confirmed to be resident in the village, and if so, name, age and sex were recorded to facilitate matching between case finding methods. Any identified SAM case not undergoing treatment was referred to the nearest outpatient center for treatment. In this study, AACF was considered exhaustive when teams were referred back to two cases already identified and all areas of the village had been visited.

**Table 2:** Case definitions used during case finding and coverage estimation [13]

SAM case	6–59 months and mid-upper arm circumference (MUAC) < 11.5 cm and/or with bi-lateral pitting edema
Recovering case	Children currently enrolled in the program but no longer meeting the anthropometric criteria of a SAM case and not yet meeting the discharge criteria for the program (MUAC > 12.4 cm for 3 consecutive visits)

#### Census method

During census case finding, the survey teams systematically visited each household in the village. Following the same household-level procedures as AACF, a household census was completed to identify all children 6–59 months of age, and all children present were evaluated using the standard case definition for SAM and recovering cases (Table 2). Census case finding was considered exhaustive when all households in the sampled village were visited.

### Statistical analysis

Sensitivity was calculated as the proportion of all SAM and recovering cases that were correctly identified as such and estimated using the Chapman modification to the Lincoln-Petersen estimator [14]. The numerator was defined as the number of cases found using each method, and a denominator was defined as the total case population (N). The total case population (N) was estimated using Eq. 1 below [9, 14] with the observed number of cases identified using each method (a, b, and c in Table 3).

**Table 3:** 2 × 2 table showing types of cases found in both samples

		Case found in census sample		
Case found in active and adaptive sample		Yes	No	*Total*
	Yes	*a*	*b*	*a + b*
	No	*c*	*x*	
	*Total*	*a + c*		*N (a + b + c + x)*

1 $$ N=\frac{\left(a+b+1\right)\times \left(a+c+1\right)}{a+1}-1\ \Big( $$

SAM treatment coverage was estimated using each case finding procedure according to current guidelines [1, 15]. To better understand the influence of the AACF sensitivity on coverage estimation, in a sensitivity analysis we calculated program coverage at varying levels of AACF sensitivity and distribution of coverage among missed cases and compared to coverage estimated using the census method.

## Results

In our study 59 SAM and recovering cases were found using AACF and 75 were found using the census method. Of those cases, 52 were found using both case finding methods, seven were found using only AACF, and 23 found only using the census method (Table 4). Three children were not found by either method. From this, we estimate the total SAM and recovering case population size across the 15 sampled villages to be 85. The sensitivity of our AACF method was 69.5% (95% CI: 59.8, 79.2) and for the census method was 91.9% (95% CI: 85.1, 98.8). The estimated SAM treatment coverage was 40.3% (95% CI 28.6, 52.0) using AACF and 34.9% (95% CI 24.7, 45.2) using the census method.

**Table 4:** Cases found during active and adaptive and census case finding

		Found in census sample		
Found in AACF sample		Yes	No	Total
	Yes	52	7	59
	No	23	3	26
	Total	75	10	85

*The 95% confidence interval for the estimate of N: (78, 92) as described in Additional file 1

In sensitivity analyses, we found that AACF yielded coverage estimates very similar to that produced using the census method when either the AACF method had high sensitivity (e.g. 90–100%) or when the program coverage in the cases missed by AACF was approximately the same as the overall coverage of 34.9% (Table 5). In our study, six out of the 23 (26%) cases not found by AACF were covered by the program. This resulted in a non-significant over-estimate of coverage in this example (40.3% with AACF vs. 34.9% with census).

**Table 5:** Estimated coverage by sensitivity of AACF and corrected for the unobserved coverage of missed cases

Coverage of missed cases (%, unobserved)	100	–	–	–	–	67.1	–	6.8	17.7	27.6	–
	90	–	–	–	–	62.0	–	11.0	20.2	28.7	–
	80	–	–	–	–	56.9	3.5	15.3	22.7	29.7	–
	70	–	–	–	–	51.9	10.2	19.5	25.2	30.8	–
	60	–	–	–	–	46.8	16.9	23.7	27.7	31.8	–
	50	–	–	–	10.3	41.8	23.5	28.0	30.2	32.9	–
	40	–	10.6	21.6	25.3	36.7	30.2	32.2	32.7	34.0	–
	30	77.5	50.6	45.2	40.3	31.7	36.9	36.4	35.2	35.0	–
	20	–	90.6	68.8	55.3	26.6	43.5	40.7	37.7	36.1	–
	10	–	–	92.4	70.3	21.5	50.2	44.9	40.2	37.1	–
		10	20	30	40	50	60	70	80	90	100
	Observed sensitivity of AACF (%)										

*Individual cells represent the estimated coverage for a given sensitivity of AACF and corrected for the unobserved distribution of coverage of missed cases. Cells without highlighting indicate an absolute difference of ±10% between the estimated coverage using the given sensitivity of AACF and distribution of coverage of missed cases vs. estimated coverage using the census case finding method (34.9%)

## Discussion

AACF has been proposed as an efficient case finding method to estimate SAM treatment coverage. In this study, we estimated the sensitivity of AACF to be 69.5% (95% CI: 59.8, 79.2), or more specifically that AACF as applied in this study correctly identified approximately 7 out of 10 SAM and recovering cases.

Field-friendly approaches for obtaining coverage estimates are now available to help nutrition program managers directly measure treatment coverage [1]. These methodologies allow for routine assessment by program staff and support community engagement through participatory methods [16] . The current operational guidance on these methods for SAM coverage estimation introduce various case finding procedures. Selection of the most appropriate procedure is necessarily context-dependent, but in practice, AACF is often considered the default method. However, as AACF is an informant-driven procedure and may yield non-representative samples, AACF case finding should be exhaustive or nearly exhaustive to produce valid SAM coverage estimates. The operational guidance specific to AACF suggests 75% sensitivity to be adequate but offers limited guidance to know exactly when this has been achieved [9]. Notwithstanding implementation of a parallel capture-recapture study to measure sensitivity, guidance suggests simply that “sampling stops only when you are sure that you have found all SAM cases in the community” and “case-finding was considered to be exhaustive when no new leads to potential cases were forthcoming and when information given by different sources (e.g., key informants and carers) identified children that had already been seen by the team” [1] . In this study, we applied a stricter definition of exhaustivity, which required teams to be referred back to cases already identified at least two times, and significant resources were made to support exhaustivity, including iterative development of a sound case definition and appropriate training of enumerators to support complete case identification.

Despite these efforts, the AACF missed a total of 26 of a potential 85 cases (30.6%), including 23 cases found using census and an estimated three cases found by neither method. The missed cases lowered AACF sensitivity below the level suggested to be acceptable by operational guidance (75%) [9]. There is little published evidence that quantifies AACF sensitivity in the context of SAM treatment; however, a report of capture-recapture studies (2003–2011), including six comparing AACF to house-to-house case finding and 17 to a central location screening method, showed sensitivities of above 75% in 20/23 (87%) studies [17]. The authors of that report acknowledge that surveys analyzed were provided from early adopters of the coverage methodology, and that subsequent results using procedures locally adapted from these early studies in other settings may not replicate these findings.

The impact of incomplete case finding (e.g. low sensitivity) on coverage estimation is not well understood, and incomplete case-finding could result in bias in either direction depending on the distribution of coverage among missed cases. Sensitivity analyses suggested that case finding should generally have a sensitivity of ≥70% in order to avoid bias of more than 10% in coverage estimation, depending on the distribution of coverage among missed cases. Program managers using AACF should consider the resources and technical capacity needed to ensure such case finding sensitivity can be achieved for valid coverage estimation and consider alternative methods (e.g. census) if necessary.

This study has a number of strengths. First, the sample size ensured greater precision to estimate sensitivity and coverage estimates. Second, careful planning was made to ensure that the five assumptions underlying the capture-recapture design were adhered to (Table 1). For example, four individual-level identifiers were collected from confirmed cases in order to allow cases in both samples to be effectively matched. A well-developed and tested local case definition ensured key informants were able to orientate enumerators towards SAM and recovering cases. The same objective case definition was applied during both case finding methods and survey enumerators were trained, standardized and supervised in anthropometric assessment, assuring a correct and equal diagnosis in both methods. To maintain independence of capture between methods, the census method systematically assessed all households in a sampled village, irrespective of case finding results using AACF the previous day. Finally, efforts were made by teams to ensure each child had an equal risk of being captured, for example by finding the child if absent from the household but known to be in the village.

Despite using the same village boundaries, avoiding known market and treatment days and encouraging carers of cases to remain at home the following day, we were unable to guarantee a perfectly “closed population” to ensure the same population was present during both samples. Violation of the assumption of a closed population meant that seven cases were found during AACF and not during the census method the following day, and an additional seven cases were absent from the village during AACF. The direction of bias in the coverage point estimate due to such missed cases depend on the distribution of coverage among these children. In future use of AACF, absent cases could be reduced by informing village authorities and carers of children aged 6–59 months to stay at home between certain hours when the survey team were to visit.

With limited operational guidance on how to define and achieve exhaustivity, future coverage assessments that use AACF should take care to develop a strong screening definition and ensure exhaustivity by all reasonable measures. This may require dedicating additional personnel to each village during case finding, communicating with village leaders prior to arriving in the village and applying strict criteria to determine when exhaustivity has been reached, such as continuing case finding until re-directed to several cases already found that day. If there is any doubt in the sensitivity of AACF being adequate, a census method, such as door-to-door sampling, might be also considered as recommended in the operational guidance [1]. In this study, 15 days were needed to complete AACF and 14.5 days to complete for census case finding. As such, a census may not present substantially greater logistical or financial burden. We further note that AACF requires the development and testing a local screening definition, and in diverse study populations, this process may need to be repeated among different sub-groups that speak different languages or represent different socio-cultural contexts. In such settings, the census method which does not require context-specific adaptations may offer a comparative efficiency. In contrast, in a large homogenous population where the same screening definition could be reasonably used for case finding across many villages, AACF may prove to be a more efficient approach than a systematic census. These results may apply to assessing coverage of SAM treatment in other rural settings, though AACF is still not recommended for assessing coverage of moderate acute malnutrition treatment (where cases are less recognizable and may not be readily identifiable by key informants), or in urban or camp settings where community cohesion may be limited and key informants may not be aware of incident cases [18].

## Conclusion

Given the impact incomplete case finding and low sensitivity can have on coverage estimation in potentially non-representative samples, adequate resources and capacity should be committed to ensure exhaustive or near exhaustive case finding.

## Supplementary information

**Additional file 1.** Supplementary Methods Appendix 1

## Data Availability

The datasets generated and/or analysed during the current study are available from the corresponding author on reasonable request.

## Abbreviations

AACF: Active and adaptive case finding
MUAC: Mid-upper arm circumference
OTP: Outpatient therapeutic program
RUTF: Ready-to-use therapeutic food
SAM: Severe acute malnutrition
SQUEAC: Semi-quantitative evaluation of access and coverage
