# Supplementary material for: Active and adaptive case finding to estimate therapeutic program coverage for severe acute malnutrition: a capture-recapture study
Source: BMC Health Serv Res. 2019 Dec 16;19:967. doi: 10.1186/s12913-019-4791-9 (PMC6916078; doi:10.1186/s12913-019-4791-9)
Supplement: Supplementary file 1 — Additional file 1. Supplementary Methods Appendix 1 [file 12913_2019_4791_MOESM1_ESM.docx]

Additional file 1

*Qualitative study to develop context-specific screening definition*

A context-specific definition of SAM was developed using qualitative methods. An interview guide, adapted from standard SQUEAC qualitative guides, was developed to ensure the key information was collected in 4 different villages. In each village, a focus group discussion (FGD), with men and another with women, and a semi-structured interview with one influential woman (e.g. a grandmother) was conducted. The villages were selected in order to cover both Hausa and Fulani communities. Hausa and Fulani speaking study staff with qualitative experience were trained to conduct the FGDs and semi-structured interviews.

The objective was to develop case definition for malnourished children and children attending the program, and identify additional vocabulary related to treatment and the programme. This included words for describing the signs and symptoms of marasmus (e.g. “emaciated”) and oedema (e.g. “swollen feet”), local understandings of malnutrition (e.g. “loss of appetite” or “when mother is breastfeeding and becomes pregnant”) and words associated with the programme (e.g. for ready-to-use therapeutic food and mid-upper arm circumference measuring tapes). Any terms that might be used to stigmatize families with malnourished children were also identified to ensure this language was avoided when case finding. Information on treatment seeking for wasting and edema was also collected which helped inform the teams on which informants may be more knowledgeable about SAM children. Reasons why people might not like children being screened were also investigated, to allow teams to account for any known barriers to screening. Finally, given malnutrition is often associated with illness, terms for common childhood illnesses such as malaria and diarrhea were also collected to help with case finding.

FGDs and semi-structured interviews were conducted using a simple guide containing 15 questions. At the end of each FGD or semi-structured interview, terms were repeated back to participants to confirm understanding. The terms were triangulated between the men and the women and across villages. The most frequently cited terms were compiled, shared and discussed with the survey team. The definition was then tested and refined in 14 additional villages over the course of 3 days. After each day (when 3-6 villages were visited) any new information was added to the case definition. This was done until no new information was found, thereby sampling to redundancy. The tried and tested case definition was then employed during case finding in the sampled villages.

*Focus group discussion/interview guide*

1. What are the most common childhood illnesses/conditions in your village?
2. Is malnutrition a problem in your village?
3. How do you know if a child is malnourished? *[List words they use to describe a malnourished child]*
4. How would you describe a child that is very thin? *[Show photos]*
5. How would you describe a child with oedema? *[Show photos]*
6. How would you describe a child that is very ill?
7. What do you do when a child is ill?
8. How do you treat a child that has malnutrition?
9. How would you describe the MUAC tape, Plumpy’nut, health centre, OTP and community health worker? *[Show tape and Plumpy’nut sachet to support]*
10. What words used to describe malnourished children (or their carers) might be offensive or unkind?
11. What are the best ways to find malnourished children in the villages?
12. Who knows about sick or malnourished children in the villages?
13. Is there any reason someone might be afraid/reluctant to go to the health centre?
14. Is there any reason that people might prevent their children from being screened/referred?

*Repeat words used to describe malnutrition/the programme back to the informants to confirm*

1. Do staff at the health centre use the same words? If not what words to they use?

*Calculating the confidence interval for N*

The approximate 95% confidence interval for the estimate of N was calculated using the following equation (9):

$$95\% CI \approx N \mp1.96 \times\sqrt{\begin{aligned} \frac{\left( a+b+1 \right)\times\left( a+c+1 \right)\times b\times\left( c-a \right)}{\left( a+1 \right)^{2} \times\left( a+2 \right)} \\ \end{aligned}}$$
